# The Framingham Heart Study, on its way to becoming the gold standard for Cardiovascular Genetic Epidemiology?

**DOI:** 10.1186/1471-2350-8-63

**Published:** 2007-10-04

**Authors:** Cashell E Jaquish

**Affiliations:** 1 Division of Prevention and Population Sciences, National Heart, Lung, and Blood Institute, National Institutes of Health, 6701 Rockledge Drive, MSC 7936 Suite 10018, Bethesda, MD 20892-7936, USA

## Abstract

The Framingham Heart Study, founded in 1948 to examine the epidemiology of cardiovascular disease in a small town outside of Boston, has become the worldwide standard for cardiovascular epidemiology. It is among the longest running, most comprehensively characterized multi-generational studies in the world. Such seminal findings as the effects of smoking and high cholesterol on heart disease came from the Framingham Heart Study. At the time of publication these were novel cardiovascular disease (CVD) risk factors, now they are the basis of treatment and prevention in the US. Is the Framingham study now on it's way to becoming the gold standard for genetic epidemiology of CVD? Will the novel genetic findings of today become the health care standards of tomorrow? The accompanying articles summarizing the results of genome-wide association studies (GWAS) give the reader a first glimpse into the possibilities.

The Framingham Heart Study, founded in 1948 to examine the epidemiology of cardiovascular disease in a small town outside of Boston, has become the worldwide standard for cardiovascular epidemiology [1,2]. It is among the longest running, most comprehensively characterized multi-generational studies in the world. Such seminal findings as the effects of smoking and high cholesterol on heart disease came from the Framingham Heart Study [3,4]. At the time of publication these were novel cardiovascular disease (CVD) risk factors, now they are the basis of treatment and prevention in the US. Is the Framingham study now on its way to becoming the gold standard for genetic epidemiology of CVD? Will the novel genetic findings of today become the health care standards of tomorrow? The accompanying articles summarizing the results of genome-wide association studies (GWAS) give the reader a first glimpse into the possibilities.

The strength of the Framingham Study has always been and will always be their extensive, well-characterized, longitudinal phenotypes. In an era of human genetics where speed is of the essence, such phenotypes are hard to come by. The recent explosion of genetic information available and low-cost genotyping technology has allowed for extensive hypothesis-free genome-wide interrogation [5,6]. Genome-wide association studies (GWAS) generally type 500,000 or more SNPs on approximately 1,000 or more individuals. Typing of an extensive number of genotypes increases the power to detect variants of small to moderate effect in a relatively small sample. However, it does create a multiple testing problem, especially in studies such as Framingham where there are hundreds of thousands of genotypes measured in thousands of individuals and associated with thousands of phenotypes. A major challenge in this GWAS endeavor will be to separate the true associations from the false positives. This is where the extensive phenotyping will be critical. Only through knowledge of related quantitative phenotypes, outcomes and genetic associations can one hope to find a hint of the underlying biological pathways leading to disease risk. This is where Framingham can become the gold standard.

Replication will be critical in determining which associations are real as we begin to see the results of multiple GWAS. A paper was recently published in *Nature *[7] providing guidelines for what constitutes replication. The authors provide an outline of points that should be addressed in an initial GWAS report; these points provide the necessary information for others to interpret and replicate the findings. The Framingham data meet all of these criteria, plus all data and results are provided to the scientific community through a full disclosure web-based resource. This enables exact replication of the Framingham findings or use of the Framingham data to replicate other GWAS findings.

The data presented in the accompanying papers are not optimal. The reports are based on a preliminary GWAS with a lower SNP density (100,000 SNPs) and a less accurate allele calling algorithm (Dynamic modeling or DM) than is the standard today [8] typed in a subset (1,345) of the Framingham cohort. However, the genotyping and allele calling algorithm were state of the art at the time they were performed and the subset consists of families, which broaden the analytic approaches that can be used. Recently genotyping of 550,000 SNPs was completed in the entire Framingham cohort (N = 9,300) [9]. Even with the limitations of the 100 K analysis the results and their implications are noteworthy and will provide information useful in interpretation of the results from the 500 K analysis.

First, because the GWAS was performed in families, results from linkage and association can be compared. Most human geneticists are abandoning linkage in favor of the latest technology-driven methods. However, linkage can provide valuable information to aid in differentiating between real associations and false positives. It was encouraging to see that two of the inflammatory markers (MCP1 concentration and CRP levels) showed concordant linkage and association peaks [9]. However, there were many cases where significant association was not accompanied by significant linkage. This is expected because of the difference in power to detect genes of small to moderate effect between the methods, and the rate of false positives.

Second, because Framingham is a cohort without disease-based ascertainment, a multitude of phenotypes can be investigated. Many of these phenotypes are correlated. This provides the opportunity to investigate pleiotropy and to begin to decipher the genetic architecture of complex diseases such as CVD. The Framingham investigators performed a first pass look at this question. They looked for overlap among the top 500 SNPs associated with phenotypes across three "working groups": glycemic/diabetes phenotypes, lipid phenotypes and obesity phenotypes. Eleven SNPs were found in more than one group, but none of these were found in all three [10-12]. These results provide intriguing areas to further investigate by actually modeling the pleiotropic effect of these variants.

Third, I think it is important to note that, despite the limitations of these data, the Framingham investigators replicated several well-established candidate gene associations. Among these were SNPs in the TCF7L2 gene and diabetes [10], SNPs in the factor VII gene and CRP gene with their respective gene products [9,13] and SNPs in the AGT gene and blood pressure [14]. In addition, several associations found in recent GWASs were replicated in the Framingham sample. Most notably is the association of coronary heart disease, CVD and coronary artery calcium [15,16] with the same region of 9p recently reported to be associated with myocardial infarction by Helgadottir *et al*. [17] and McPherson *et al*. [18].

The analysis of such a large volume of data and presentation of results is a daunting task. The Framingham investigators have done a commendable job in getting the results out and providing the data for other investigators to use. In the process they have entered the "gold rush" to stake claims on GWAS results [19]. Although this initial task was daunting, what lies ahead is even more so. Results such as these as they stand are valuable in their own right. However, they are invaluable when interpreted within the context of other genetic and environmental risk factors. It is in this next step of delving beyond the p-values where Framingham can set the gold standard. The Framingham Heart Study has meticulously collected the information necessary to decipher the genetic and environmental risk factors for cardiovascular disease and they have the expertise to do so. Only through such a meticulous approach can we provide an integrated, holistic view of health and disease. The world wide scientific community will be looking to the Framingham Heart Study to set the gold standard once again, this time in the genomic era.

## Pre-publication history

The pre-publication history for this paper can be accessed here:


